# Innate Immune Responses of *Drosophila melanogaster* Are Altered by Spaceflight

**DOI:** 10.1371/journal.pone.0015361

**Published:** 2011-01-11

**Authors:** Oana Marcu, Matthew P. Lera, Max E. Sanchez, Edina Levic, Laura A. Higgins, Alena Shmygelska, Thomas F. Fahlen, Helen Nichol, Sharmila Bhattacharya

**Affiliations:** 1 Space Biosciences Division, NASA Ames Research Center, Mountain View, California, United States of America; 2 Carl Sagan Center, SETI Institute, Mountain View, California, United States of America; 3 Lockheed Martin Exploration & Science, NASA Ames Research Center, Mountain View, California, United States of America; 4 Silicon Valley Campus of Carnegie Mellon University, NASA Ames Research Center, Mountain View, California, United States of America; 5 Department of Anatomy and Cell Biology, University of Saskatchewan, Saskatoon, Saskatchewan, Canada; Columbia University, United States of America

## Abstract

Alterations and impairment of immune responses in humans present a health risk for space exploration missions. The molecular mechanisms underpinning innate immune defense can be confounded by the complexity of the acquired immune system of humans. *Drosophila* (fruit fly) innate immunity is simpler, and shares many similarities with human innate immunity at the level of molecular and genetic pathways. The goals of this study were to elucidate fundamental immune processes in *Drosophila* affected by spaceflight and to measure host-pathogen responses post-flight. Five containers, each containing ten female and five male fruit flies, were housed and bred on the space shuttle (average orbit altitude of 330.35 km) for 12 days and 18.5 hours. A new generation of flies was reared in microgravity. In larvae, the immune system was examined by analyzing plasmatocyte number and activity in culture. In adults, the induced immune responses were analyzed by bacterial clearance and quantitative real-time polymerase chain reaction (qPCR) of selected genes following infection with *E. coli*. The RNA levels of relevant immune pathway genes were determined in both larvae and adults by microarray analysis. The ability of larval plasmatocytes to phagocytose *E. coli* in culture was attenuated following spaceflight, and in parallel, the expression of genes involved in cell maturation was downregulated. In addition, the level of constitutive expression of pattern recognition receptors and opsonins that specifically recognize bacteria, and of lysozymes, antimicrobial peptide (AMP) pathway and immune stress genes, hallmarks of humoral immunity, were also reduced in larvae. In adults, the efficiency of bacterial clearance measured *in vivo* following a systemic infection with *E. coli* post-flight, remained robust. We show that spaceflight altered both cellular and humoral immune responses in *Drosophila* and that the disruption occurs at multiple interacting pathways.

## Introduction

Spaceflight alters both cellular and humoral immune responses in humans. Moreover, impaired immunity may present a risk for manned short and long-term exploration missions [1], [2]. In humans, spaceflight affects cell-mediated immunity by altering the production and distribution of leukocytes and the activity of natural killer cells, phagocytic neutrophils and macrophages (reviewed in [3]). In addition, spaceflight affects humoral immunity including the production of interferon and interleukins [4]–[9].

Alterations in the immune system can also be induced *via* mimicking the microgravity environment of spaceflight using analogous environments on Earth. For example, following extended bed rest, humans have low antibody production and increased tumor necrosis factor alpha levels [10], while longterm hindlimb unloading in mice increases susceptibility to infections ([11], [12], reviewed in [13]). The molecular mechanisms underlying changes in immunity are poorly understood in part because the human innate and acquired immune systems are complex and overlap [14] and in part because the stress of spaceflight has compound effects.

The fruit fly is a useful model to tease out these mechanisms because it has a highly conserved innate immunity but lacks a traditional cell-mediated acquired immunity and the vast adaptability of the human immune system [15]–[20]. However, the immune responses of *Drosophila* can be ‘primed’ by exposure to certain pathogens, which activate a set of intra- and extra-cellular pathogen recognition molecules under complex control [21], [22]. This priming effect makes flies more resistant to a subsequent infection [23].

Fruit fly innate immunity includes humoral and cellular factors. The cellular responses by blood cells (hemocytes) include the recognition, phagocytosis and encapsulation of microbes [24], [25]. The humoral factors induce hemolymph coagulation, melanization and the synthesis of antimicrobial peptides (AMPs) [26], [27].

AMPs are expressed constitutively in barrier epithelia and are upregulated upon infection [28]. AMP synthesis is also induced in the fat body both by systemic infection ([29], [30], reviewed in [31]) and by stress *via* the FOXO pathway [32].

Fungi and Gram-positive bacteria activate a set of AMPs *via* the Toll regulatory pathway while Gram-negative bacteria activate AMPs *via* the immune deficiency (Imd) pathway [17], [33], [34]. Although the induction of AMPs was initially thought to be specific to either the Toll or Imd pathway, there is ample evidence that the two pathways overlap [34]–[37]. Both Toll and Imd pathways affect the production of AMPs through the nuclear relocation of dorsal, dif and relish, which can act as transcriptional regulators. In addition to Toll and Imd, the less well-characterized JAK/STAT pathway (Janus kinase signal transducers and activators of transcription) also plays a key role in immunity ([38], [39], reviewed in [40]).

Since the human immune system is complex, we used the *Drosophila* model to tease out the effects of spaceflight on the simpler innate immune system of the fruit fly, focusing on molecular pathways that perform similar functions in the human immune system. In larvae we found reduced cellular phagocytosis of *E. coli* and reduced expression levels of genes encoding hemocyte maturation markers and phagocytosis receptors. Genes involved in humoral immunity (pattern recognition receptors, AMPs, lysozymes and turandot) were also significantly downregulated. The adult flies retained the ability to clear a systemic *E. coli* infection despite short-term alterations of AMP induction.

## Results

### Larval Immune Responses

#### 1. Larvae reared in space are smaller and have fewer plasmatocytes

**Figure 1:**
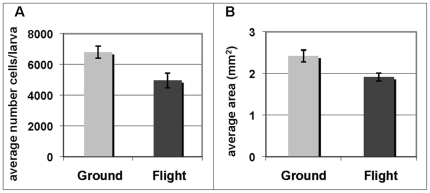
Space-reared larvae have fewer plasmatocytes and are smaller. A. The average number of plasmatocytes per larva was significantly reduced in flight vs ground (*p*-value <0.008, n = 20). B. Larvae reared in space were significantly smaller than ground-reared larvae (*p*-value <0.0085, n = 10).

Late 3^rd^ instar larvae within a 6-hour developmental window, characterized by the clearing of gut contents prior to pupation, were used to examine the immune responses of larvae. The average number of plasmatocytes per animal was lower in space-reared larvae (Fig. 1A) than analogous ground-reared larvae. We could not directly convert this quantity to the number of plasmatocytes per unit volume of hemolymph because the space-reared larvae were smaller (Fig. 1B) and we observed that they contained less fat body than ground controls, which made it difficult to assess the remaining volume of the hemocoel occupied by hemolymph. We therefore examined other indicators of the cellular immune response, specifically the phagocytic capacity of plasmatocytes.

#### 2. Plasmatocyte phagocytic activity is impaired in space-reared larvae

**Figure 2:**
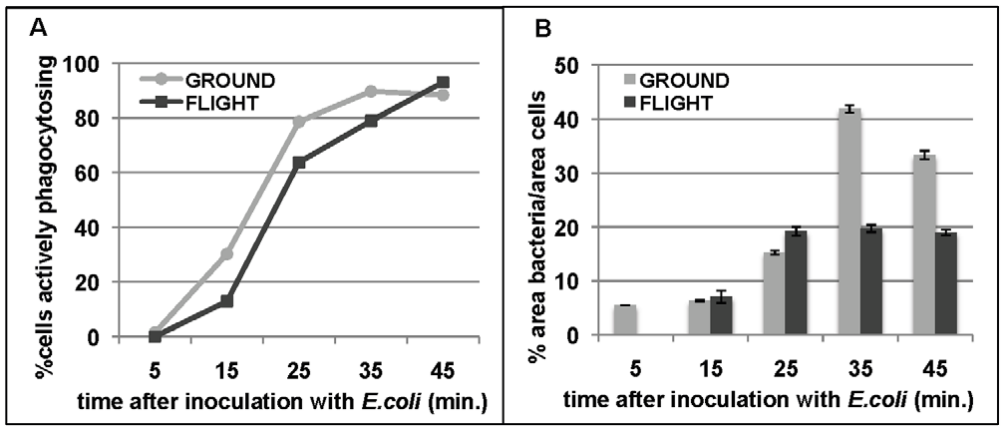
Spaceflight alters phagocytosis. A. The number of actively phagocytosing cells is reduced by spaceflight. At 15, 25, and 35 minutes, a significantly lower number of cells engulfed *E. coli* in hemocyte cultures from spaceflight larvae. The calculated *p*-values were 0.32, 2.16E-07, 3.38E-05, 3.86E-08, and 5.8E-04 for the 5, 15, 25, 35 and 45-minute time points respectively. The data points are tight and give calculated standard error values in the 10^−2^ range, not visible on the graph. B. The phagocytic capacity of larval plasmatocytes is stunted. The number of Alexa594-labeled *E. coli* engulfed by larval plasmatocytes in culture was compared in samples from flight and ground up to 45 minutes after infection. Cells from both space- and ground-reared larvae had similar levels of activity up to 25 minutes post-infection, while after 35 minutes space larvae plasmatocytes had significantly reduced phagocytic capacity.

We quantified the ability of GFP-labeled plasmatocytes in cell cultures to phagocytose Alexa594-labeled *E. coli* over time by analyzing both the number of cells capable of active phagocytosis as well as the amount of bacteria taken up by active cells. We found that hemocyte cultures from larvae that developed from egg to 3^rd^ instar in space had a significantly smaller percentage of cells engulfing bacteria at the early 15, 25 and 35 minutes post-infection (Fig. 2A). The calculated *p*-values were 0.32, 2.16E-07, 3.38E-05, 3.86E-08, and 5.8E-04 for the 5, 15, 25, 35 and 45-minute time points, respectively. This suggests that the onset of phagocytosis may be delayed in space-reared larvae. Active cells from both flight and ground showed a similar level of phagocytosed bacteria up to 25 minutes post-infection (Fig. 2B). However, at 35 and 45 minutes post-infection, the number of *E. coli* inside plasmatocytes isolated from space-reared larvae plateaued, whereas plasmatocytes from the ground-reared larvae continued to engulf bacteria, suggesting a reduction in phagocytic capacity in the space-reared larvae.

Taken together, the reduction in the number of phagocytosing cells and in the number of bacteria taken up by active cells, indicate that the cellular phagocytosis aspect of the immune system is downregulated in space-reared larvae. We next used microarray analysis of gene expression levels in 3^rd^ instar larvae to identify possible genes that may cause this impairment of phagocytosis related to spaceflight.

#### 3. The population of mature hemocytes is affected in space-reared larvae

Genome arrays showed that many genes associated with the process of cellular immunity and phagocytosis were significantly downregulated after spaceflight (Table 1), including markers of hemocyte development and maturation.

**Table 1:** The expression of genes involved in hemocyte maturation and pathogen recognition and binding is downregulated in spaceflight larvae.

Gene	Symbol	Probe #	Adjusted *p*-value	Up or Down	Fold change
**Hemocyte Maturation**					
Hemese	He	1640654	1.9×10^−8^	D	0.29
lethal (3) malignant blood neoplasm	l(3)mbn	1633919	8.5×10^−4^	D	0.53
Peroxidasin	Pxn	1640097	3.8×10^−3^	D	0.72
PDGF- and VEGF-related factor 2	Pvf2	1630642	1.5×10^−2^	D	0.69
lozenge	lz	1633299	3.5×10^−2^	D	0.74
***E. coli*** ** binding and phagocytosis**					
Scavenger receptor class C, type I	Sr-CI	1631821	3.2×10^−5^	D	0.63
Scavenger receptor class C, type IV	Sr-CIV	1624817	3.0×10^−5^	D	0.56
Down syndrome cell adhesion molecule	Dscam	1637619	3.0×10^−2^	D	0.79
Galactose-specific C-type lectin	Lectin-galC1	1633378	5.5×10^−3^	D	0.60
Thioester containing protein I	TepI	1637734	1.9×10^−8^	D	0.25
Thioester containing protein II	TepII	1630067	1.5×10^−2^	D	0.76
**Pattern Recognition Receptors**					
Gram-negative bacteria binding protein 3	GNBP3	1635470	1.8×10^−4^	D	0.67
Peptidoglycan recognition protein SB1	PGRP-SB1	1636490	6.0×10^−7^	D	0.31
Peptidoglycan recognition protein SA	PGRP-SA	1628884	5.3×10^−6^	D	0.62
Peptidoglycan recognition protein LA	PGRP-LA	1625486	1.3×10^−3^	D	0.74
Peptidoglycan recognition protein LF	PGRP-LF	1633145	1.9×10^−2^	D	0.68
Peptidoglycan recognition protein SC2	PGRP-SC2	1629118	3.0×10^−3^	U	1.41

Among the major categories of genes that were affected in larvae that developed in microgravity were the genes that mark the differentiation and maturation of plasmatocytes (l(3)mbn, Pxn, Pvf2,) and crystal cells (lz); genes involved in *E. coli* binding and phagocytosis; and the pattern recognition receptors GNBP3 and PGRPs which activate the humoral pathways. Gene expression level in spaceflight samples compared to ground controls is expressed as fold change. The statistical significance of the gene changes is shown by the adjusted *p*-value. The false discovery rate (FDR) cut-off was less than 0.05 for all genes.

Third instar larvae contain three populations of hemocytes: active circulating cells that are specified in the embryonic head mesoderm [41], cells that form, differentiate and mature in the lymph gland [42], [43] and a sessile population of hemocytes associated with the epidermis and imaginal discs [44], [45]. Circulating hemocytes are mature and functional at all larval stages, the lymph gland contains pre-prohemocytes, prohemocytes and maturing hemocytes that are not active in the larva, but are released into circulation at metamorphosis and contribute to the adult hemocyte population [46], and the sessile precursors are the source of mature lamellocytes released into circulation upon infection [47]. The specification and maturation of hemocytes are determined during development by the expression of markers particular to each cell lineage.

We analyzed the expression of markers associated with the stages of hemocyte development and maturation at the 3^rd^ instar and compared their expression level during spaceflight with that of ground controls. We found that only the markers of mature hemocytes were differentially expressed between ground and flight larvae, while it appeared that the markers of the early stages of hemopoiesis were not affected by spaceflight (Table 1). The markers *serpent* (*srp*, CG3992) and *odd skipped* (*odd*, CG3851), present in all hemocyte precursors, was not changed by spaceflight. The non-proliferative, quiescent medullary zone of the lymph gland, containing undifferentiated prohemocytes [43], [48], is marked by the presence of *unpaired3* (*upd3*, CG33542) whose expression also did not change. For precursors of individual cell lineages, the expression of *viking* (CG16858) and *cg25C* (CG16858) (collagen markers of immature plasmatocytes and crystal cells [49], [50]), *glial cells missing gcm* (CG12245) and *gcm2* (CG3858) (markers of plasmatocyte specification [51]) and *collier* (CG10197, the early marker of undifferentiated crystal cells and lamellocytes in the posterior signaling center [52]), all remained unaltered by spaceflight. This suggests that the immature cell population was unaffected. However, the gene *hemese* (*he*, CG31770), expressed in hemocytes of all stages at the 3^rd^ instar [53] was downregulated. This indicated changes in the total cell population and prompted us to ask whether the genes that specify the mature hemocyte lineages (plasmatocytes, crystal cells and lamellocytes) may be downregulated. *Misshapen* (CG16973), a marker for mature lamellocytes [43], [54] remained unchanged, suggesting that the lamellocyte population was not affected as expected since lamellocyte differentiation occurs only upon infection [47]. The expression of *serrate* (CG6127) and *Notch* (CG3936), that initiates crystal cell fate, did not change significantly, but *lozenge* (*lz*, CG1689), the marker for the final stage of mature crystal cells, was highly downregulated. Similarly, PDGF- and VEGF Receptor related (*Pvr*, CG8222) which specifies the plasmatocyte lineage was unchanged, but its ligand *Pvf2* (CG13780) involved in plasmatocyte differentiation [55] and *peroxidasin* (*pxn*, CG12002) and *l(3)mbn* (CG12755), associated with mature plasmatocytes, were significantly downregulated. These findings indicate that while the immature hemocyte population was unchanged in larvae, the population of mature crystal cells and plasmatocytes was affected by spaceflight. Since the maturation of hemocytes is required for their function, it is possible that it could contribute to the overall reduction in the number of actively phagocytosing cells. Given that the active cells from space-reared larvae also engulfed lower numbers of bacteria than the cells from ground control larvae, we next looked at whether this partial reduction in phagocytic capacity could be due to an impaired ability of plasmatocytes to recognize bacteria.

#### 4. Pattern recognition molecules are downregulated in spaceflight larvae

We observed a significant downregulation of pattern recognition receptor genes in microarrays from space-reared larvae. *Sr-CI* (CG4099) and *Sr-CIV* (CG3212) (Scavenger receptors Class C, type I and IV), of which a Sr-CI is a macrophage integral membrane protein responsible for *E. coli* binding [56], [57], *Dscam* (Down syndrome cell adhesion molecule, CG17800), expressed in hemocytes and involved in efficient phagocytosis of *E. coli* [58] and *lectin-galC1* (CG9976), promoting the agglutination of *E. coli* cells and their association with phagocytosing macrophages [59], all had lower level of expression. The TEP opsonins (thioester-containing proteins) are involved in the recognition and binding of bacteria and specifically mediate cellular phagocytosis (reviewed in [60]). We found that *TepI* (CG18096) and *TepII* (CG7052) were significantly downregulated. TepI is expressed in hemocytes upon induction with *E. coli* [61], and TepII is required for efficient phagocytosis of *E. coli* [62]. In contrast, the expression of *TepIII* (CG7068), which is required for the phagocytosis of the Gram-positive *Staphylococcus aureus*, but not that of *E. coli* [60], did not change. The immune-related receptor *croquemort* (*crq*, CG4280) was also upregulated, but it is involved in the phagocytosis of apoptotic bodies, not bacteria [63], [64]. The expression of the phagocytosis receptor *eater* (CG6124) [65] did not change after spaceflight, and nor did that of other genes that affect phagocytosis through vesicle trafficking and interaction with the cytoskeleton [62], [66].

Taken together, these data suggest a reduced capacity of plasmatocytes to mature into cells that effectively recognize and bind bacteria and may explain the reduced phagocytic activity observed in space-reared larvae.

We have also found a significant downregulation of genes involved in receptor-mediated pattern recognition and binding of pathogens: GNBP3 (Gram-negative bacteria binding protein, CG5008), and members of the PGRP (peptidoglycan recognition protein) class of receptors [67], [68], [69]. None of these affect the phagocytosis of *E. coli* (see discussion), but they significantly affect the downstream activation of humoral immune responses and the expression of AMPs. Moreover, the only gene upregulated was PGRP-SC2, which is an antagonist of Imd induction, consistent with a suppression of humoral immunity [70]. Therefore we next examined the effect of spaceflight on humoral immunity.

#### 5. The constitutive expression of genes involved in humoral immunity is downregulated in space-reared larvae

**Figure 3:**
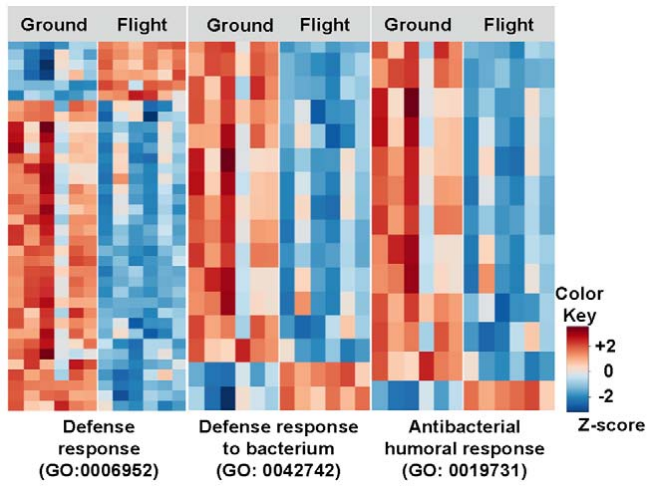
Humoral responses are downregulated in spaceflight larvae. Gene Ontology (GO) categories of defense response show altered gene expression in flight larvae compared to ground-reared larvae: 30 out of the 37 classified as defense response are downregulated in flight. These include the majority of response to bacteria and in particular humoral response genes. Six experimental repeats of each of the samples (flight and ground) were used to extract RNA for microarray analysis and are represented in individual columns in the figure. Rows represent levels of individual gene expression in each of the experimental repeats. Data is shown following Z-score transformation. Red colors indicate Z-scores >0 (above mean), blue colors indicate Z-scores <0 (below mean). The gene names can be found in Tables 1 and 2.

Microarray analysis and classification according to over-represented Gene Ontology categories showed that the humoral immune defense pathway was downregulated in 3^rd^ instar larvae exposed to spaceflight (Fig. 3). The significantly altered genes, classified by function as shown in Table 2, include the AMP response genes *Imd* (CG5576) and *spatzle* (*spz*, CG6134), which were both downregulated. Interestingly, *ect4* (CG34373) and the serine proteases *SPE* (*Spatzle-Processing Enzyme*, CG16705) and *spheroide* (CG9675), required for activating Toll but upstream of *spz* expression [71], [72], were upregulated. All the target AMP genes that showed differential expression between spaceflight and ground (*metchnikowin* CG8175, *attacins* CG10146, CG4740, CG7629, *drosocin* CG10816 and *drosomycin* CG10810) were downregulated. Besides the AMP pathway, the lysozyme genes *LysX* (CG9120) and *LysP* (CG9116) [73], the stress-responsive turandot genes *TotC* (CG31508) and *TotA* (CG31509) [74], [75] and *Thor* (CG8846), involved in the response to Gram-positive bacteria [76] were also downregulated.

**Table 2:** The constitutive expression of humoral immunity genes is altered in spaceflight larvae.

Gene Category	Symbol	Probe #	Adjusted *p*-value	Up or Down	Fold Change
**AMP response**					
immune deficiency	imd	1637332	1.3×10^−2^	D	0.80
spatzle	spz	1641068	1.3×10^−2^	D	0.72
Spatzle-processing enzyme	SPE	1634848	4.7×10^−3^	U	1.49
spheroide	spheroide	1624578	2.8×10^−3^	U	1.90
Ect4	Ect4	1632067	2.7×10^−2^	U	1.44
Calpain-A	CalpA	1628628	1.9×10^−3^	U	1.49
dorsal	dl	1623415	2.1×10^−4^	D	0.60
Metchnikowin	Mtk	1627613	3.9×10^−7^	D	0.16
Attacin-D	AttD	1631475	8.7×10^−7^	D	0.42
Attacin-C	AttC	1641419	6.2×10^−5^	D	0.24
Drosomycin	Drs	1635189	3.5×10^−4^	D	0.57
Attacin-A	AttA	1625124	5.4×10^−4^	D	0.14
Drosocin	Dro	1631697	6.6×10^−3^	D	0.70
**Other humoral factors**					
Thor	Thor	1635900	8.5×10^−3^	D	0.78
**Stress factors**					
Turandot A	TotA	1635549	8.7×10^−7^	D	0.12
Turandot C	TotC	1639323	4.3×10^−4^	D	0.36
**Bacterial cell wall lysis**					
Lysozyme X	LysX	1632720	4.6×10^−11^	D	0.14
Lysozyme P	LysP	1636767	7.3×10^−4^	D	0.52
**Melanization**					
Black cells	Bc	1635494	3.2×10^−11^	D	0.29

Specific classes of genes involved in the humoral immune response that were altered by spaceflight included: the AMP response pathway, the stress factors turandot A and C, the lysozyme genes and the melanization gene black cells. Although some of the genes upstream of AMPs were upregulated, the target AMP effectors were all downregulated. The statistical significance of gene changes is shown by the adjusted *p*-value at an FDR threshold of less than 0.05.

Since the larvae (both control and space-reared) were not challenged by experimental infection, AMP, Lys and Tot expression should not have been induced in either group. Therefore the downregulation we see probably reflects a reduction in the constitutive expression level of these humoral factors.

Due to the observed changes in both cellular and humoral aspects of larval immunity, and to the fact that the hemocytes in the larval lymph gland contribute to the adult immunity, we next examined the cellular and humoral immune responses of adults.

### Adult Immune Responses

#### 1. Bacterial clearance in adults remains robust after spaceflight

**Figure 4:**
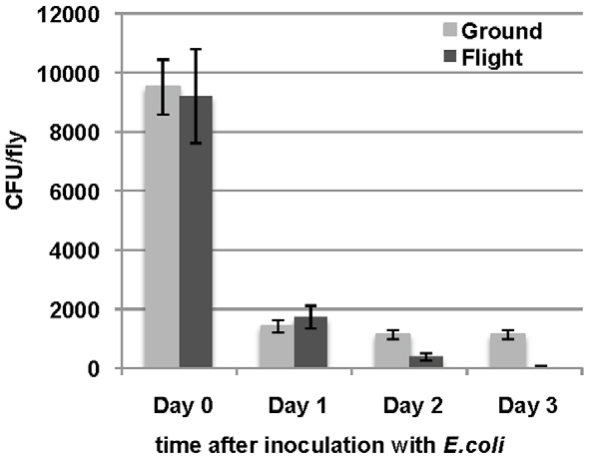
The ability to clear bacterial infections is maintained in adult flies after spaceflight. Adults were infected with *E. coli* HB101 immediately after return from spaceflight. The overall capacity to clear the bacterial infection was measured by quantifying the live bacteria (CFU) remaining in the animal up to 3 days following infection. Flight adults cleared bacteria as effectively as controls immediately after flight, and more efficiently at days 2 and 3 post infection (*p-*value <0.05).

The ability to clear a hemolymph *E. coli* infection was compared between control adults and adults infected 24 hours after return from flight. The adult flies returned from spaceflight cleared the majority of the infection within the first day after the infection and were as effective as the control flies (Fig. 4). By the third day the flies from spaceflight had completely cleared all the bacteria, while the ground control flies still had a level of approximately 1,000 colony-forming units (CFU) per fly.

We next examined in more detail the contribution of humoral pathway to the bacterial clearance observed. We analyzed the constitutive level of genes involved in humoral immunity using microarrays analysis of RNA from uninfected adults, and the induced humoral response after a systemic infection with *E. coli* using quantitative real-time polymerase chain reaction (qPCR).

#### 2. Microarrays do not reveal changes in the constitutive expression of immunity genes in adults recovered from spaceflight

Adult females collected on the first day after landing (F1), that had completed a full generation (developed and hatched) in space, were used for RNA extraction and analysis. The level of RNA expression was compared to that of ground controls removed from their cultures at the same time. It should be noted that fewer flight adults survived than control adults (data not shown).

Microarray analysis of gene expression in uninfected F1 adults showed no significant changes in the expression of genes from either the cellular or humoral response pathways. Since the constitutive immunity seemed to be unaltered in the uninfected adults that survived spaceflight, we next examined the capacity of the adult flies to mount an induced immune response upon bacterial challenge.

#### 3. Spaceflight alters the induced response to *E. coli* infection

**Figure 5:**
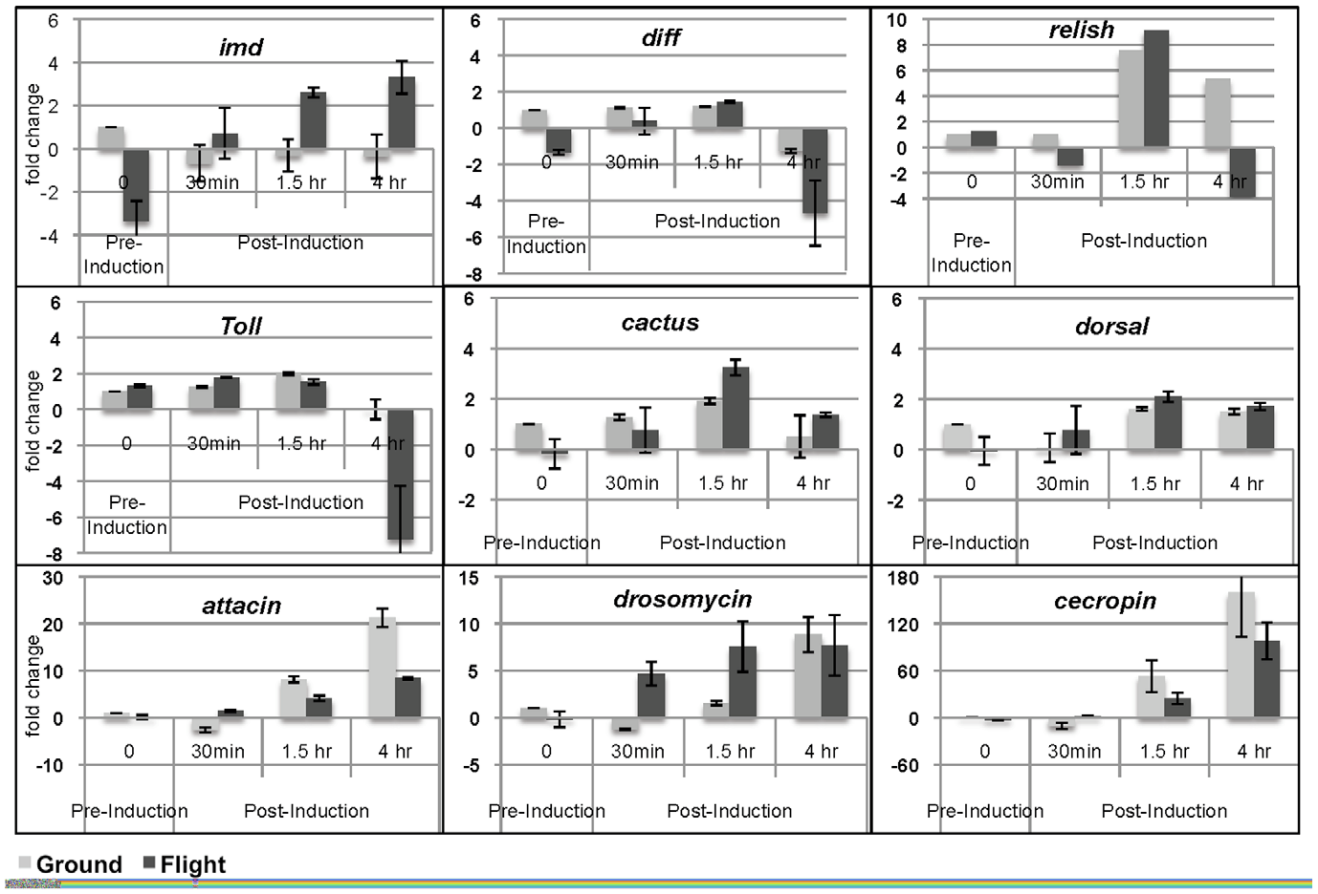
The humoral response to *E. coli* infection in adults after spaceflight. The time course of gene expression after bacterial infection was compared between flight and ground-reared adult females using qPCR. Data was averaged from two experimental samples, with the exception of relish. Mock- infected flight RNA levels were normalized against ground control to obtain the pre-infection level, and all time course points for ground and flight were normalized against their respective pre-infection level.

The expression of genes in the Imd pathway was measured by qPCR analysis of space and ground-reared adults in a PBS mock-infected control and within 30 minutes, 1.5 and 4 hours after systemic infection with *E. coli* HB101. We measured the expression of genes in the Imd and Toll pathways that regulate the expression of AMP target genes. We found that the initial, pre-induction level of *Imd* in PBS controls was lower in flight adults than in ground-reared adults (Fig. 5). However, at 1.5 and 4 hours after infection, the expression of *Imd* was significantly upregulated in flight compared to ground adults. We next looked at downstream regulators of the Imd pathway, the genes *relish* (CG11992) and *dif* (dorsal-related immunity factor, CG6794). The expression of both genes was similar in ground and spaceflight adults up to 1.5 hours after infection but at 4 hours post-infection the expression of *relish* (CG11992) and *dif* was significantly reduced in flies returned from spaceflight. Furthermore, the expression of *attacin*, a target of relish and dif, was also significantly downregulated in flight compared to ground at 4 hours. Since *attacin* transcription is cross-regulated by Toll acting through relish [77], we also looked at expression level of members of the Toll pathway.

The expression of *Toll* (*Tl*, CG5490) was unaltered in flight pre-induction (prior to infection), but was downregulated at 4 hours by more than 4 fold. The main downstream effectors of Toll, *cactus* (CG5848) and *dorsal* (*dl*, CG6667), were essentially unchanged between flight and ground-reared adults. The AMP *cecropinA1* (CG1365) showed similar induction of expression between ground and flight. In contrast, the expression of *drosomycin* (CG10810) was induced earlier after infection and at higher levels in flight than in ground adults. Therefore, spaceflight affects the short-term *E. coli*-induced expression of AMPs at several levels, likely through both Imd and Toll pathways, however the adults are still able to effectively clear the *E. coli* bacterial infection.

## Discussion

The overall goal of this study was to identify those aspects of *Drosophila* innate immunity that were affected by spaceflight. We found that spaceflight altered cellular and humoral immune responses in larval and adult *Drosophila* at the physiological and gene expression levels. We observed a reduction in the number and activity of larval plasmatocytes after spaceflight. While we could not directly determine that the reduced number of plasmatocytes was due to fewer cells or to a smaller volume of hemolymph in space-reared larvae, this also coincided with a downregulation of markers of mature cells, which may contribute to a reduction in the mature cell population. The populations of lamellocytes, preprohemocytes, prohemocytes and early hemocytes appeared to be normal since their markers were unchanged. However, the downregulation of *hemese* (*he*), as well as of markers of mature plasmatocytes (*Pxn*) and crystal cells (*lz*) is consistent with reduced numbers of mature cells. While there were insufficient larvae to statistically confirm our initial observation that crystal cell numbers were reduced, we did find that the gene *Black cells* (*Bc*), involved in the melanization process by crystal cells [27], was highly downregulated.

There is an interesting parallel between our observations in *Drosophila* and what has been reported in humans following spaceflight. In our experiment, several markers of mature plasmatocytes and crystal cells were downregulated. Similarly, in humans there is evidence that the differentiation of both macrophages and neutrophils was inhibited [2], [78], [79]. During three shuttle flights immature band neutrophils were observed in peripheral blood of astronauts that were not seen before or after the flight [80]. In humans, the neutrophil is the primary phagocytic cell that fights bacterial infections and a primary line of defense in the innate immune response. Following short-duration spaceflight on the Shuttle, the population of granulocytes was consistently elevated in human peripheral blood [9], [81], [82], [83], and chemotaxis was significantly increased during 8, 9 and 14 day space shuttle missions [80]. There was also a shift towards less differentiated CD8+ T lymphocytes [9]. Our data confirms that even short-term spaceflight affects the fundamental processes of cellular and humoral immunity as well as the maturation of immune cells.

We also showed that the functional ability of plasmatocytes to phagocytose bacteria was delayed, and could not be sustained in space-reared larvae. While the delay may be partially due to the maturation of hemocytes, the block to full phagocytic capacity also coincided with the downregulation of Sr-CI, Dscam and Tep opsonins, crucial to the efficient phagocytosis of *E. coli*. This strongly suggests that the binding and phagocytosis of bacteria are affected by spaceflight. While TepI and TepII are expressed constitutively at low levels, and only upregulated upon immune challenge [61], we found that spaceflight affects the constitutive expression of all these classes of pattern recognition molecules. We also checked the RNA expression levels of genes involved in actin binding and regulation, vesicle transport and the other classes of genes shown by Stroschein-Stevenson et al. to reduce the phagocytosis of *E. coli* [62], and we found that only 4 genes were altered after spaceflight. Of these, only *gartenzwerg* (*garz*, CG8487) has an assigned function and is known to be involved in clathrin-independent pinocytosis. This suggests that pinocytosis may be affected in spaceflight, while most other cellular functions are probably intact. Our results indicate that the phagocytic response of plasmatocytes after spaceflight was initiated at normal levels but it could not be sustained, likely through a reduced ability to bind and internalize bacteria. Taken together, the reduced number of cells, downregulation of cellular immune genes and impaired phagocytosis indicate that the cellular arm of the immune defense is affected by spaceflight at multiple levels.

We also found that the constitutive larval humoral immunity was impaired from the first step of bacterial recognition by PGRPs all the way to the production of AMPs. GNBP3 recognizes β-glucans on the surface of fungi and can activate the Toll pathway [84], [85], and members of the PGRP class of receptors recognize and bind either Gram-positive or Gram-negative bacteria and activate either Toll or Imd [67], [68], [69]. PGRP-LA (CG32042) and PGRP-SA (CG11709) do not affect the phagocytosis of *E. coli* [86], but PGRP-SA is a main activator of the Toll pathway. PGRP-SB1 (CG9681) is expressed in gut upon infection [87], and PGRP-LF (CG4437) has been shown to downregulate the Imd pathway [86].

Consistent with this, microarray analysis showed reduced levels of *imd* and of the target genes *attacins* and *drosocin*. Although the serine proteases *SPE* and *spheroide*, activators of Toll through the cleavage of *spatzle* [72], as well as *ect4*, whose homologue in *C. elegans* has been shown to activate Toll [88], were upregulated, the level of target AMPs *drosomycin* and *metchnikowin* was reduced. A possible explanation is that the downstream expression of *spatzle*, which was downregulated, is epistatic to the activity of these genes and could be the limiting factor for AMP production. AMP expression is induced in fat body by systemic infection, in gut by ingestion and in epithelium by abrasion, while barrier epithelia express some AMPs constitutively [30]. The AMP expression we measured is most likely constitutive, since the larvae were not experimentally infected.

Besides the constitutive downregulation of AMPs in larvae, other responses to spaceflight also occurred in the absence of bacterial challenge. For example, the lysozyme gene LysX that is important in the defense against ingested bacteria [89] and the stress response genes *TotA* and *TotC*, [75] were all highly downregulated. In contrast, *TotM*, which is primarily upregulated upon septic injury [90], was unchanged. These changes in gene expression suggest that spaceflight impacts immunity at a fundamental level even prior to microbial infection.

Since both the humoral gene expression and the phagocytosis of *E. coli* were lower in larvae, we examined these parameters in adults. Overall, ground and flight adults were able to clear *E. coli* effectively after landing. To dissect out the effects of spaceflight on the humoral immune response following *E. coli* infection, we analyzed the expression of genes in humoral response pathways using qPCR. The two-fold downregulation of *Imd* in PBS-injected flight flies as compared to ground-reared flies may reflect a lower constitutive level of Imd protein. Following infection, the ground-reared adults show a level of *Imd* induction similar to that reported previously [91]. In contrast, at 4 hours, flight flies showed about 3 fold more *Imd* than ground-reared adults and 6 fold more relative to pre-induction levels. We suggest that this more vigorous upregulation represents a ‘priming’ event induced by spaceflight.

The only observed indicator consistent with the higher capacity to clear bacteria after 48 hours was the increase in *Imd*, which may result in higher AMP production long term. Spaceflight may also trigger other pathways that could enhance bacterial clearance. One of these is the JAK/STAT pathway that is known to play a key role in immunity ([38], [39] and reviewed in [40]). However, in our experiment, the ligand for the Jak-STAT pathway *outstretched* (*os*, CG5993) remained unchanged, and so did the downstream genes *domeless* (*dome*, Os receptor, CG14226), *hopscotch* (*hop*, the human Jak2 homologue, CG1594) and *marelle* (STAT92E homologue, CG4257). This indicates that Jak/STAT signaling most likely did not contribute to the observed spaceflight-induced alterations in immunity. However, given that the expression of genes in the *Toll* pathway was affected by spaceflight, it would be valuable to look at the phenotypes following infection with a Gram-positive bacterium. Only *E. coli* was used in the experiment presented here, due to spaceflight logistics and the restricted number of flies permitted within the payload mass and volume constraints.

We observed that more pupae died in space than on ground (data not shown) and so it is possible that larvae with poor immunity did not survive metamorphosis. The importance of functioning plasmatocytes in immune surveillance during the pupal phase has recently been demonstrated by targeting plasmatocytes for apoptosis [92], however, other factors may also have increased pupal lethality. Either way, the population of adult flies that survived spaceflight may represent a select population that could efficiently clear bacteria.

Overall, our data indicate that spaceflight alters both the cellular and the humoral immune responses of *Drosophila* in several fundamental ways that resemble the suppression of innate immunity observed in humans. If similar changes in gene expression are found in humans exposed to long-term microgravity, this will have important implications for extended manned space missions. Our findings show that the immune disruption occurs at several levels, including the host-pathogen interaction, and imply that maintaining immune integrity during the microgravity environment of spaceflight would require a concerted regulation of all these mechanisms.

## Methods

### Fly line and culture

The *Drosophila melanogaster* Gal4-UAS transgenic line expressing two copies of eGFP under the control of the hemolectin promoter [93] was used in all experiments. This line expresses GFP in all plasmatocytes and has no other phenotype. Flies were grown on semi-defined medium (1% agar, 4% brewer's yeast, 2% yeast extract, 2% peptone, 3% sucrose, 6% glucose, 0.05% MgSO_4_, 0.05% CaCl_2_ and tegosept) to which 0.5% blue food dye was added to facilitate the recognition of late 3^rd^ instar larvae at wandering stage by the absence of colored food in the gut.

### Experimental conditions

Flies for both the flight and the ground control experiments were transferred from fresh cultures to fly kits 24 hours in advance of the onset of the experiment. Each fly kit, consisted of a polycarbonate fly cassette with a 2 ml food tray held in a ventilated aluminum container. These kits were originally developed by the European Space Agency and used previously for *Drosophila* experiments in space [94], [95], and were subsequently modified by us for use in this experiment [96]. Ten virgin females and 5 males were housed in each fly cassette. Ten kits (5 with flies and 5 empty) flew on the space shuttle and 10 remained on the ground. The fly kits were placed in stowage foam on the shuttle middeck, at ambient temperature and in total darkness. The identical procedure was followed for the ground control flies. For both ground and flight experiments, on day 6 the food trays containing eggs and larvae were transferred to the 5 empty cassettes and fresh food was given to the original flies. The temperature and relative humidity in the spaceflight and ground payloads were monitored minute-by-minute using a HOBO data logger (Onset Computer Corporation). Values were tracked in real time and paralleled for the ground control in the OES (Orbital Environmental Simulator) at the Kennedy Space Center. Environmental data from flight and ground experiments were provided by the operations team as part of the routine shuttle flight logistics. The average temperature on the flight deck was 23.5°C, ranging between 21.71°C and 25.56°C. The ground control average temperature was 22.5°C, with a minimum of 19.42°C and a maximum of 25.56°C. The relative humidity was 27.2% on the shuttle deck and 25.4% on ground.

### Hemocyte counts

Counting larval hemocytes followed previously published protocols [97], [98]. Briefly, late 3^rd^ instar larvae at wandering stage were rinsed in ice-cold Schneider's insect medium (Sigma) supplemented with complete Mini Protease Inhibitor Cocktail (Sigma). For each hemocyte count, blood from three 3^rd^ instar larvae was mixed with 60 µl fresh medium and a hemocytometer was used to count cells from a 20 µl aliquot. Hemocytes were identified using a Zeiss AxioskopII equipped with a GFP filter, served by an Optronics camera and Magnafire imaging software.

### Larval body size

3^rd^ instar larvae were fixed in 4% paraformaldehyde, 50% acetone for 2 hours, followed by 3 hours in 4% paraformaldehyde in PBS, rinsed for 1 hour in PBS and stored at −20°C. The area of each larva was determined with Image ProPlus software (Media Cybernetics) and the average size was determined from 10 larvae for each condition, spaceflight samples were compared to controls.

### Phagocytosis Assay

Third instar larvae were thoroughly washed with 70% ethanol, 50% dilution of regular Clorox bleach (∼5% sodium hypochlorite) and sterile water and wiped on filter paper. Larval hemocytes were isolated and cultured in SFBS (Schneider's medium supplemented with 12% fetal bovine serum). Pooled blood from 5 larvae was added to 400 µl ice-cold culture medium with no antibiotic, and incubated on a rocker at 25°C for 20 minutes.

Hemocytes were treated with 0.5 µl of a 20 mg/ml Alexa Fluor 594-labeled *E. coli* bacterial suspension (Invitrogen, E-22370) and incubated at 25°C for 5, 15, 25, 35 or 45 minutes. The cells in 200 µl aliquots were distributed in 10-well glass slides (Precision Scientific) and the cells were allowed to adhere for 30 minutes, in the dark. The cells were gently washed with PBS followed by the addition of 0.5 µl 0.2% trypan blue in PBS to each well to quench non-phagocytosed bacteria. The wells were covered with a 20×50 mm coverslip and cells were imaged under a 40x objective Zeiss Axioskop II with rhodamine and GFP filters, using the Optronics MagnaFire software. Each time point was done in duplicate and 10 areas were imaged from each replicate (20 images and approximately 50–60 plasmatocytes total per time point. The area (µm^2^) occupied by internalized bacteria in each plasmatocyte was determined at each time point using automated image analysis, supported by Image ProPlus software. Standard errors were calculated for each time point, and the Student's t-test was used to determine the *p*-values for significant differences between flight and ground data sets.

### Bacterial clearance assay

Adult flies from flight and control cassettes were injected in the abdominal cavity with 50 nanoliters of an *E. coli* HB101 strain at 2×10^8^ cells/ml in PBS, using a Picospritzer III (Parker Instrumentation). Only female flies were used, all male adults were provided to other participating scientists. Mock-infected flies were injected with PBS alone. At 0 (pre-infection) and 24, 48 and 72 hours after infection, 3 flies from each time point were ground in 200 µl LB with 1% Triton X-100, and serial dilutions were plated on LB plates containing 50 µg/ml streptomycin. Colonies were counted after 24-hour incubation at 37°C and results were reported in CFU (colony forming units)/fly, averaged from 3 repeat plates.

### Assay of gene induction following bacterial infection

Three females adult flies collected on the first day of landing and 3 ground control flies were collected at 4 time points: pre-infection (PBS injected flies), within 30 minutes of infection, and at 1.5 and 4 hours after *E. coli* infection as described above. RNA was isolated using a Qiagen RNeasy Kit (Qiagen), and reverse transcribed into cDNA using the cDNA Archive Kit (Applied Biosystems). A 7500 Real-Time Polymerase Chain Reaction System (Applied Biosystems) was used to amplify selected genes, following standard protocols. Inventoried Applied Biosystems sets of primers and FAM/TAMRA labeled probes were Dm01821460 (*metchnikowin*), Dm01810797 (*Dif*), Dm02134843 (*relish)*, Dm02151201 (*Toll)*, Dm01807756 (*cactus*), Dm01810803 (*dorsal*), Dm01845288 (*Imd*), Dm02151531 (*spaetzle*), Dm01822006 (*drosomycin,* specific to CG10810), and custom made probes for *attacinA*, *cecropinA1*, and the ribosomal RNA gene *Rib15A* as control were obtained from IDT (Integrated DNA Technologies).

The fold change in RNA level was estimated using the 2^-ΔΔC^_T_ method [99], [100]. Mock- infected flight RNA levels were normalized against ground control to obtain the pre-infection level, and all time course points thereafter, for ground and flight, were normalized against their respective pre-infection level.

### Microarray sample preparation

Larval RNA for microarrays was prepared from 6 sets of 50 mid 3^rd^ instar larvae reared in microgravity and collected on the day of landing of the space shuttle, and the same number of control larvae raised on ground. The space shuttle flight had a total duration of 12 days and 18.5 hours. Adult RNA was prepared from 3 sets of 20 adult females each that emerged during the flight and within 4 hours of landing (Flight Day 1) and from adult females from the corresponding ground control cassettes (Ground Day 1). The original parental flies were removed. Given the known generation time at 24°C, the adults that emerged in space (F1) would have been no more than 2 days old.

All RNA was isolated using the Qiagen RNeasy Kit. RNA samples were processed and hybridized to *Drosophila 2.0* Affymetrix arrays using standard Affymetrix protocols by the Protein and Nucleic Acid Facility, Beckman Center, Stanford University. Six sets of larval arrays and 3 sets of adult arrays were used to provide repeats for statistical validation.

### Microarray Data Analysis

Quality of microarrays: RNA degradation levels were verified by ordering individual probes in a probe set by location relative to the 5′ end of the targeted RNA molecule. Probe intensities were averaged across all genes by probe number.

Preprocessing: Background correction was performed using MAS (Microarray Analysis Suite) software (Affymetrix Inc). Quantile normalization and pmonly and liwong summarization was used. Multiple probe sets targeting the same gene were filtered according to their largest variability to emphasize the most informative probe sets.

Test for differential expression: Differentially expressed genes were identified by fitting the moderated t-test linear model to the data (separately for each gene). Bayesian smoothing was used to control for the number of arrays. To control the False Discovery Rate (FDR) during multiple testing, the FDR criterion introduced by Benjamini and Hochberg [101] was applied to *p*-values. FDR adjusted *p*-values are reported. The significance threshold used for FDR was 5% (0.05).

Clustering: For identifying gene pathways and networks affected by spaceflight, differentially expressed genes and genes of interest (specific Gene Ontology (GO) categories) were sorted using hierarchical clustering. The Pearson correlation distance metric was used. In heatmap figures (Fig. 3) we used Z-score, a measure of distance in standard deviations from the mean [102]. A Z score of 0 has the same raw value as the mean, and Z scores of 1.0 and −1.0 are exactly one standard deviation above and below the mean, respectively.

Lists of differentially expressed genes were compiled using conditional hyper-geometric testing and computing *p*-values for overrepresentation of genes in all GO terms.

Implementation: Gene expression analysis was implemented using the free software Bioconductor version 2.11.1.

All genomic data is MIAME compliant and the raw data has been deposited in the GEO database under the accession number GSE23880.
